# Gene network interconnectedness and the generalized topological overlap measure

**DOI:** 10.1186/1471-2105-8-22

**Published:** 2007-01-24

**Authors:** Andy M Yip, Steve Horvath

**Affiliations:** 1 Department of Mathematics, National University of Singapore, 2, Science Drive 2, Singapore 117543, Singapore; 2 Department of Human Genetics and Department of Biostatistics, University of California, Los Angeles, CA 90095, USA

## Abstract

**Background:**

Network methods are increasingly used to represent the interactions of genes and/or proteins. Genes or proteins that are directly linked may have a similar biological function or may be part of the same biological pathway. Since the information on the connection (adjacency) between 2 nodes may be noisy or incomplete, it can be desirable to consider alternative measures of pairwise interconnectedness. Here we study a class of measures that are proportional to the number of neighbors that a pair of nodes share in common. For example, the topological overlap measure by Ravasz *et al*. [1] can be interpreted as a measure of agreement between the *m *= 1 step neighborhoods of 2 nodes. Several studies have shown that two proteins having a higher topological overlap are more likely to belong to the same functional class than proteins having a lower topological overlap. Here we address the question whether a measure of topological overlap based on higher-order neighborhoods could give rise to a more robust and sensitive measure of interconnectedness.

**Results:**

We generalize the topological overlap measure from *m *= 1 step neighborhoods to *m *≥ 2 step neighborhoods. This allows us to define the *m*-th order generalized topological overlap measure (GTOM) by (i) counting the number of *m*-step neighbors that a pair of nodes share and (ii) normalizing it to take a value between 0 and 1. Using theoretical arguments, a yeast co-expression network application, and a fly protein network application, we illustrate the usefulness of the proposed measure for module detection and gene neighborhood analysis.

**Conclusion:**

Topological overlap can serve as an important filter to counter the effects of spurious or missing connections between network nodes. The *m*-th order topological overlap measure allows one to trade-off sensitivity versus specificity when it comes to defining pairwise interconnectedness and network modules.

## Background

We consider undirected, unweighted biological networks that can be represented by a symmetric adjacency matrix *A *= [*a*_*ij*_]. The adjacency *a*_*ij *_between nodes *i *and *j *equals 1 if the nodes are connected and 0 otherwise. For notational convenience, we set the diagonal elements to 1. While the adjacency matrix considers each pair of genes in isolation, topological overlap considers each pair of genes in relation to all other genes in the network. More specifically, genes are said to have high topological overlap if they are connected to roughly the same group of genes in the network (i.e. they share the same neighborhood). To calculate the topological overlap for a pair of genes, their connections with all other genes in the network are compared. If the 2 nodes connect to the same group of other nodes, then they have a high 'topological overlap'. Here we study the properties of the topological overlap measure (TOM) and propose a generalization that enriches TOM's sensitivity to longer ranging connections between nodes.

There is empirical evidence that two substrates having a higher overlap are more likely to belong to the same functional class than substrates having a lower topological overlap [1-5]. Such a finding prompts the question whether a measure of topological overlap based on higher-order neighborhoods would lead to a more sensitive and robust measure of interconnectedness. In this paper, we generalize the topological overlap measure by incorporating information from higher-order neighborhoods and show that it leads to a definition of larger modules. Specifically, the *m*-th order topological overlap measure is constructed by (i) counting the number of *m*-step neighbors that a pair of nodes share and (ii) normalizing it to take a value between 0 and 1. The resulting node similarity measure is a measure of agreement between the *m*-step neighborhoods of 2 input nodes. Such a measure can be applied in a number of ways, for instance, ranking the genes, similarity search, prediction based on *k*-nearest neighbors, multi-dimensional scaling and module identification by clustering.

## Results

The algebraic definition of the topological overlap measure can be found in Eq. (7) in the Methods section. Here we provide a more intuitive set theoretic interpretation of the topological overlap measure. Our generalization of the TOM is motivated by the observation that Eq. (7) can be expressed as

tij={|N1(i)∩N1(j)|+aijmin⁡{|N1(i)|,|N1(j)|}+1−aijif i≠j1if i=j.     (1)
 MathType@MTEF@5@5@+=feaafiart1ev1aaatCvAUfKttLearuWrP9MDH5MBPbIqV92AaeXatLxBI9gBaebbnrfifHhDYfgasaacH8akY=wiFfYdH8Gipec8Eeeu0xXdbba9frFj0=OqFfea0dXdd9vqai=hGuQ8kuc9pgc9s8qqaq=dirpe0xb9q8qiLsFr0=vr0=vr0dc8meaabaqaciaacaGaaeqabaqabeGadaaakeaacqWG0baDdaWgaaWcbaGaemyAaKMaemOAaOgabeaakiabg2da9maaceqabaqbaeqabiGaaaqaamaalaaabaWaaqWaaeaacqWGobGtdaWgaaWcbaGaeGymaedabeaakiabcIcaOiabdMgaPjabcMcaPiabgMIihlabd6eaonaaBaaaleaacqaIXaqmaeqaaOGaeiikaGIaemOAaOMaeiykaKcacaGLhWUaayjcSdGaey4kaSIaemyyae2aaSbaaSqaaiabdMgaPjabdQgaQbqabaaakeaacyGGTbqBcqGGPbqAcqGGUbGBcqGG7bWEdaabdaqaaiabd6eaonaaBaaaleaacqaIXaqmaeqaaOGaeiikaGIaemyAaKMaeiykaKcacaGLhWUaayjcSdGaeiilaWYaaqWaaeaacqWGobGtdaWgaaWcbaGaeGymaedabeaakiabcIcaOiabdQgaQjabcMcaPaGaay5bSlaawIa7aiabc2ha9jabgUcaRiabigdaXiabgkHiTiabdggaHnaaBaaaleaacqWGPbqAcqWGQbGAaeqaaaaaaOqaaiabbMgaPjabbAgaMjabbccaGiabdMgaPjabgcMi5kabdQgaQbqaaiabigdaXaqaaiabbMgaPjabbAgaMjabbccaGiabdMgaPjabg2da9iabdQgaQjabc6caUaaaaiaawUhaaiaaxMaacaWLjaWaaeWaaeaacqaIXaqmaiaawIcacaGLPaaaaaa@7C7D@

where *N*_1_(*i*) denotes the set of direct neighbors of *i *excluding *i *itself and |·| denotes the number of elements (cardinality) in its argument. The quantity |*N*_1_(*i*) ∩ *N*_1_(*j*)| measures the number of common neighbors that nodes *i *and *j *share whereas |*N*_1_(*i*)| gives the number of neighbors of *i*. The topological overlap *t*_*ij *_assumes a minimal value of 0 if there is no direct linkage between the two nodes and if they share no common direct neighbors. It assumes a maximum value of 1 if there is a direct link between the two nodes and if one set of direct neighbors is a subset of the other. The fact that *t*_*ij *_is bounded between 0 and 1 is used in the definition of the topological overlap based dissimilarity measure (see Eq. 4).

### Generalizing TOM to *m*-step neighborhoods

By denoting *N*_*m*_(*i*) (with *m *> 0) the set of nodes (excluding *i *itself) that are reachable from *i *within a path of length *m*, i.e.,

*N*_*m*_(*i*) := {*j *≠ *i*|dist(*i*, *j*) ≤ *m*}     (2)

where dist(*i*, *j*) is the geodesic distance (shortest path distance) between *i *and *j*, we obtain a very natural generalization of the TOM, which reads as follows

tij[m]={|Nm(i)∩Nm(j)|+aijmin⁡{|Nm(i)|,|Nm(j)|}+1−aijif i≠j1if i=j.     (3)
 MathType@MTEF@5@5@+=feaafiart1ev1aaatCvAUfKttLearuWrP9MDH5MBPbIqV92AaeXatLxBI9gBaebbnrfifHhDYfgasaacH8akY=wiFfYdH8Gipec8Eeeu0xXdbba9frFj0=OqFfea0dXdd9vqai=hGuQ8kuc9pgc9s8qqaq=dirpe0xb9q8qiLsFr0=vr0=vr0dc8meaabaqaciaacaGaaeqabaqabeGadaaakeaacqWG0baDdaqhaaWcbaGaemyAaKMaemOAaOgabaGaei4waSLaemyBa0Maeiyxa0faaOGaeyypa0ZaaiqabeaafaqabeGacaaabaWaaSaaaeaadaabdaqaaiabd6eaonaaBaaaleaacqWGTbqBaeqaaOGaeiikaGIaemyAaKMaeiykaKIaeyykICSaemOta40aaSbaaSqaaiabd2gaTbqabaGccqGGOaakcqWGQbGAcqGGPaqkaiaawEa7caGLiWoacqGHRaWkcqWGHbqydaWgaaWcbaGaemyAaKMaemOAaOgabeaaaOqaaiGbc2gaTjabcMgaPjabc6gaUjabcUha7naaemaabaGaemOta40aaSbaaSqaaiabd2gaTbqabaGccqGGOaakcqWGPbqAcqGGPaqkaiaawEa7caGLiWoacqGGSaaldaabdaqaaiabd6eaonaaBaaaleaacqWGTbqBaeqaaOGaeiikaGIaemOAaOMaeiykaKcacaGLhWUaayjcSdGaeiyFa0Naey4kaSIaeGymaeJaeyOeI0Iaemyyae2aaSbaaSqaaiabdMgaPjabdQgaQbqabaaaaaGcbaGaeeyAaKMaeeOzayMaeeiiaaIaemyAaKMaeyiyIKRaemOAaOgabaGaeGymaedabaGaeeyAaKMaeeOzayMaeeiiaaIaemyAaKMaeyypa0JaemOAaOMaeiOla4caaaGaay5EaaGaaCzcaiaaxMaadaqadaqaaiabiodaZaGaayjkaiaawMcaaaaa@8231@

We define the matrix *T*^[*m*] ^= [tij[m]
 MathType@MTEF@5@5@+=feaafiart1ev1aaatCvAUfKttLearuWrP9MDH5MBPbIqV92AaeXatLxBI9gBaebbnrfifHhDYfgasaacH8akY=wiFfYdH8Gipec8Eeeu0xXdbba9frFj0=OqFfea0dXdd9vqai=hGuQ8kuc9pgc9s8qqaq=dirpe0xb9q8qiLsFr0=vr0=vr0dc8meaabaqaciaacaGaaeqabaqabeGadaaakeaacqWG0baDdaqhaaWcbaGaemyAaKMaemOAaOgabaGaei4waSLaemyBa0Maeiyxa0faaaaa@34E5@] as the *m-th order generalized topological overlap measure *(GTOM*m*). Thus, GTOM*m *measures the agreement of the *m*-step neighborhoods between 2 nodes. When *m *= 1, this definition reduces to the original TOM in Eq. (7).

We find it convenient to define the zeroth order GTOM0 as the adjacency matrix, i.e. *T*^[0] ^≡ *A*. Since *T*^[*m*] ^is symmetric and non-negative, *T*^[*m*] ^can be considered as a *similarity *measure [6]. To turn *T*^[*m*] ^into a dissimilarity measure for use in clustering, we make use of the fact that tij[m]
 MathType@MTEF@5@5@+=feaafiart1ev1aaatCvAUfKttLearuWrP9MDH5MBPbIqV92AaeXatLxBI9gBaebbnrfifHhDYfgasaacH8akY=wiFfYdH8Gipec8Eeeu0xXdbba9frFj0=OqFfea0dXdd9vqai=hGuQ8kuc9pgc9s8qqaq=dirpe0xb9q8qiLsFr0=vr0=vr0dc8meaabaqaciaacaGaaeqabaqabeGadaaakeaacqWG0baDdaqhaaWcbaGaemyAaKMaemOAaOgabaGaei4waSLaemyBa0Maeiyxa0faaaaa@34E5@ is bounded by 1. The generalized topological overlap-based dissimilarity measure is defined by

dijT,[m]=1−tij[m].     (4)
 MathType@MTEF@5@5@+=feaafiart1ev1aaatCvAUfKttLearuWrP9MDH5MBPbIqV92AaeXatLxBI9gBaebbnrfifHhDYfgasaacH8akY=wiFfYdH8Gipec8Eeeu0xXdbba9frFj0=OqFfea0dXdd9vqai=hGuQ8kuc9pgc9s8qqaq=dirpe0xb9q8qiLsFr0=vr0=vr0dc8meaabaqaciaacaGaaeqabaqabeGadaaakeaacqWGKbazdaqhaaWcbaGaemyAaKMaemOAaOgabaGaeeivaqLaeiilaWIaei4waSLaemyBa0Maeiyxa0faaOGaeyypa0JaeGymaeJaeyOeI0IaemiDaq3aa0baaSqaaiabdMgaPjabdQgaQbqaaiabcUfaBjabd2gaTjabc2faDbaakiabc6caUiaaxMaacaWLjaWaaeWaaeaacqaI0aanaiaawIcacaGLPaaaaaa@46AB@

### Predicting essential proteins in a Drosophila protein network

Knock-out experiments in lower organisms (e.g. yeast, fly, worm) have shown that essential proteins tend to be more highly connected than non-essential proteins [7-9]. To illustrate the biological usefulness of the proposed interconnectedness measures, we set out to test the ability of GTOM to predict essential proteins using a Drosophila (fly) protein-protein interaction network from the BioGrid Database [10]. In our version of the database, the largest connected component was comprised of 2294 proteins with 21217 pairwise interactions. Knock-out experiments had implicated 282 essential proteins.

To illustrate the use of GTOM*m*, we will show that proteins that are highly interconnected with an essential protein have an increased chance of being essential as well. Toward this end, we make use of the following terminology. A GTOM*m *neighborhood of size *S *around node *i *is defined as the set GTOMhoodS[m]
 MathType@MTEF@5@5@+=feaafiart1ev1aaatCvAUfKttLearuWrP9MDH5MBPbIqV92AaeXatLxBI9gBaebbnrfifHhDYfgasaacH8akY=wiFfYdH8Gipec8Eeeu0xXdbba9frFj0=OqFfea0dXdd9vqai=hGuQ8kuc9pgc9s8qqaq=dirpe0xb9q8qiLsFr0=vr0=vr0dc8meaabaqaciaacaGaaeqabaqabeGadaaakeaacqWGhbWrcqWGubavcqWGpbWtcqWGnbqtcqWGObaAcqWGVbWBcqWGVbWBcqWGKbazdaqhaaWcbaGaem4uamfabaGaei4waSLaemyBa0Maeiyxa0faaaaa@3BF5@(*i*) of *S *genes with highest GTOM value with *i*. For example, node *j *is in the size *S *= 1 GTOM*m *neighborhood around node *i *if it has the highest topological overlap tij[m]
 MathType@MTEF@5@5@+=feaafiart1ev1aaatCvAUfKttLearuWrP9MDH5MBPbIqV92AaeXatLxBI9gBaebbnrfifHhDYfgasaacH8akY=wiFfYdH8Gipec8Eeeu0xXdbba9frFj0=OqFfea0dXdd9vqai=hGuQ8kuc9pgc9s8qqaq=dirpe0xb9q8qiLsFr0=vr0=vr0dc8meaabaqaciaacaGaaeqabaqabeGadaaakeaacqWG0baDdaqhaaWcbaGaemyAaKMaemOAaOgabaGaei4waSLaemyBa0Maeiyxa0faaaaa@34E5@ across all nodes. For simplicity, we ignore ties and define GTOMhoodS[0]
 MathType@MTEF@5@5@+=feaafiart1ev1aaatCvAUfKttLearuWrP9MDH5MBPbIqV92AaeXatLxBI9gBaebbnrfifHhDYfgasaacH8akY=wiFfYdH8Gipec8Eeeu0xXdbba9frFj0=OqFfea0dXdd9vqai=hGuQ8kuc9pgc9s8qqaq=dirpe0xb9q8qiLsFr0=vr0=vr0dc8meaabaqaciaacaGaaeqabaqabeGadaaakeaacqWGhbWrcqWGubavcqWGpbWtcqWGnbqtcqWGObaAcqWGVbWBcqWGVbWBcqWGKbazdaqhaaWcbaGaem4uamfabaGaei4waSLaeGimaaJaeiyxa0faaaaa@3B80@(*i*) as the set of genes that directly link to node *i *(irrespective of the neighborhood size *S*).

Since essential proteins may participate in the same pathway, it is biologically plausible that the GTOM*m *neighborhoods of an initial essential protein contain essential proteins as well. Below, we show that for a fixed neighborhood size *S *and a fixed essential protein, the GTOM2 neighborhood contains a higher proportion of essential proteins than the corresponding GTOM0 or GTOM1 neighborhoods. This provides indirect empirical evidence that the proposed higher order GTOM measure (*m *= 2) outperforms the standard GTOM measure (*m *= 1) in this application.

**Figure 1:**
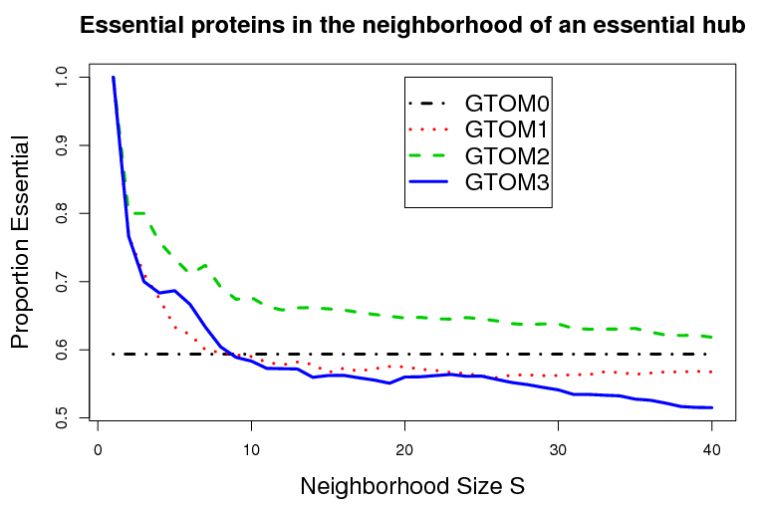
**Proportions of essential proteins among the *S *proteins that are most highly interconnected with a given essential hub protein in the Drosophila protein-protein interaction network**. For a given neighborhood size *S *(*x*-axis) we averaged the results over 30 essential hub proteins (*y*-axis). The black horizontal line (GTOM0) represents the average proportion of essential proteins among the directly linked neighbors (adjacency = 1) of an essential hub protein.

Specifically, we considered the neighborhoods around each of the 30 essential proteins with highest nodal degrees (referred to as essential hub proteins). Since on average the GTOM0 neighborhoods of these essential hub proteins contain 40 proteins, we considered the following neighborhood sizes *S *= 1,..., 40. For each of the essential hub proteins, we determined the GTOM*m *neighborhood for *m *= 0,1,2,3. For a given neighborhood of size *S*, we determined the proportion of essential proteins. Next we averaged these proportions across the 30 essential hub proteins. Figure 1 reports the average proportion of essential proteins (*y*-axis) versus different neighborhood sizes (*x*-axis).

As can be seen from Figure 1, GTOM2 performs better than the other measures in this application involving a relatively sparse network. For example, when considering neighborhoods comprised of 10 proteins (rank 10) based on GTOM0, GTOM1, GTOM2, GTOM3 the proportion of essential proteins is given by 0.59, 0.59, 0.68, and 0.58, respectively. We find that neighborhood analysis with GTOM2 leads to significantly better results than GTOM0 (Wilcoxon *p*-value = 0.034), GTOM1 (*p*-value = 0.015) and GTOM3 *p*-value = 0.02).

### Module detection in a yeast co-expression network

There is evidence that genes and their protein products carry out cellular processes in the context of functional modules [11]. Thus, an important task in biological network analysis is to identify groups, or 'modules', of densely interconnected genes. Here we focus on module identification methods that are based on using a node dissimilarity measure in conjunction with a clustering method. Further, we assume that the nodes in a network module have high topological overlap with their neighbors. A review of alternative module detection methods is beyond the scope of this article, see e.g. [12-21].

**Figure 2:**
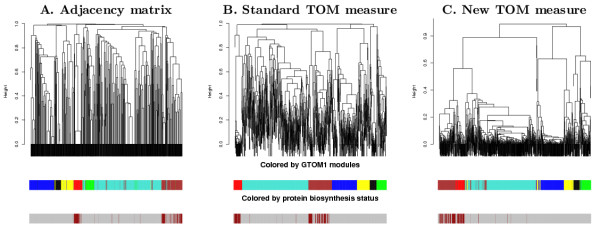
**Yeast network modules and protein biosynthesis genes for different GTOM*m***. **A**. The adjacency matrix (GTOM0). **B**. Standard Ravasz *et al*.'s TOM (GTOM1). **C**. Our new generalized TOM (GTOM2). In each column, the top row shows the dendrogram obtained by applying the average linkage hierarchical clustering to the corresponding GTOM dissimilarity, the middle row shows the color bar ordered by the corresponding dendrogram but colored by the module assignment with respect to the TOM measure in **B**, the bottom shows the color bar ordered by the corresponding dendrogram but colored in dark red if the gene belongs to the class 'protein biosynthesis'. The modules defined by the TOM are the branches of the dendrogram in **B **at the cutoff 0.95. Almost all protein biosynthesis genes are grouped together by the proposed new TOM measure whereas the other two measures tend to distribute the class over two modules. The modules defined by GTOM2 are more pronounced in the sense that they are separated by larger distances.

To demonstrate the usefulness of the GTOM dissimilarity measures for module detection, we apply the proposed measures to gene co-expression networks constructed based on a microarray dataset recording gene expression levels during different stages of cell cycle in yeast [22]. Because the transcriptional response of cells to changing conditions involves the coordinated co-expression of genes encoding interacting proteins, studying co-expression patterns can provide insights into the underlying cellular processes [23,24]. As detailed in the Methods section, the co-expression network was constructed by thresholding the absolute pair-wise (Pearson) correlation coefficient between the expression profiles. We cluster the genes into modules using the average linkage hierarchical clustering with different choices of dissimilarity measures. Modules correspond to branches of the resulting clustering tree. While there is evidence that this clustering procedure leads to biologically meaningful modules in several applications [1-5,25], we do not claim that this clustering method is optimal. Since our interest lies in the performance of topological overlap based dissimilarity measures but not the clustering procedure, comparing different clustering procedures is beyond the scope of this article. In our applications, modules correspond to branches of a hierarchical clustering dendrogram [6]. Figure 2 shows the modules (as branches of the dendrogram) detected by applying the average linkage hierarchical clustering with 3 different similarity measures: The adjacency matrix (GTOM0), Ravasz *et al*.'s TOM (GTOM1) and a generalized TOM (GTOM2) presented in the Results section. Genes that belong to the functional class 'protein biosynthesis' are grouped together when the GTOM2 measure is used. However, they are separated into two distinct subgroups if GTOM0 or GTOM1 are used. This suggests that GTOM2 is a more sensitive measure for detecting the higher order connections between the nodes in this large module. Thus membership in the protein biosynthesis module is more robust when neighborhoods of step size 2 is used for measuring topological overlap.

For the sake of brevity, we only present our analysis of the protein biosynthesis module in this methodological paper. Since the protein biosynthesis pathway is relatively large, it makes sense to use a relatively sensitive dissimilarity measure (GTOM2) since it favors the discovery of large modules. However, when considering a functional category that involves few genes, it would be better to use a dissimilarity measure with higher specificity (GTOM0 or GTOM1) since it favors the discovery of smaller modules. A more detailed biological analysis of a related yeast co-expression network can be found in [3].

#### Comparing GTOM*m *to the correlation coefficient

Since the absolute value of the Pearson correlation coefficient is widely used for clustering gene expression profiles, we compare it here to the GTOM measures. Specifically, we consider the following class of correlation based dissimilarities:

dijC,[p]=1−|ρij|p.     (5)
 MathType@MTEF@5@5@+=feaafiart1ev1aaatCvAUfKttLearuWrP9MDH5MBPbIqV92AaeXatLxBI9gBaebbnrfifHhDYfgasaacH8akY=wiFfYdH8Gipec8Eeeu0xXdbba9frFj0=OqFfea0dXdd9vqai=hGuQ8kuc9pgc9s8qqaq=dirpe0xb9q8qiLsFr0=vr0=vr0dc8meaabaqaciaacaGaaeqabaqabeGadaaakeaacqWGKbazdaqhaaWcbaGaemyAaKMaemOAaOgabaGaee4qamKaeiilaWIaei4waSLaemiCaaNaeiyxa0faaOGaeyypa0JaeGymaeJaeyOeI0IaeiiFaWhcciGae8xWdi3aaSbaaSqaaiabdMgaPjabdQgaQbqabaGccqGG8baFdaahaaWcbeqaaiabdchaWbaakiabc6caUiaaxMaacaWLjaWaaeWaaeaacqaI1aqnaiaawIcacaGLPaaaaaa@47A3@

Here, *ρ*_*ij *_is the (Pearson) correlation coefficient between the expression profiles of *i *and *j*. Setting *p *= 1 yields the absolute correlation coefficient which is widely used for clustering genes. Setting *p *> 1 has the effect of emphasizing larger values of |*ρ*_*ij*_| while deemphasizing the smaller ones. We consider *p *= 6 since we find that the resulting distance is highly related to GTOM1 in the yeast dataset. Such a setting has also been used in [26] for functional annotation.

**Figure 3:**
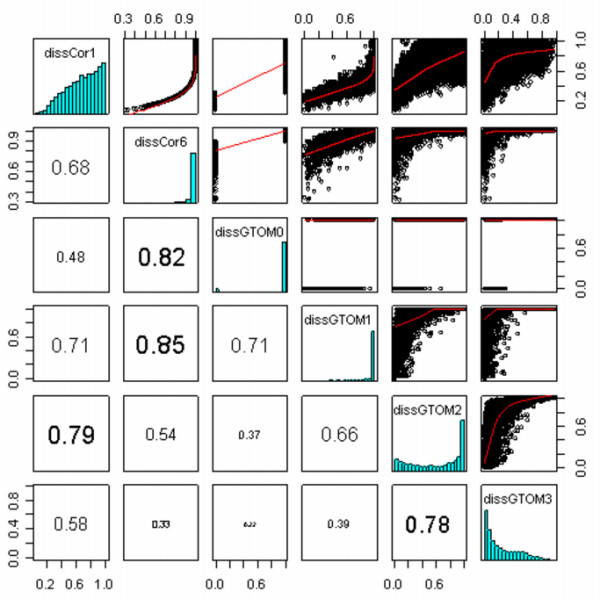
**Pair-wise scatter plots between different GTOM*m *dissimilarity measures**. The upper triangular panel shows the scatter plots, the lower triangular panel shows the corresponding Pearson correlation coefficients, the diagonal panel shows the frequency distributions of the dissimilarities. Correlation-based dissimilarities *d*^C,[*p*] ^are denoted by dissCor*p*. GTOM-based dissimilarities *d*^T,[*m*] ^are denoted by dissGTOM*m*. Note that dissGTOM0 (= 1 - ADJ) takes on binary values for the unweighted network.

Figure 3 shows the relationship between six dissimilarity measures, GTOM-based *d*^T,[*m*] ^for *m *= 0,1,2,3 and correlation-based *d*^C,[*p*] ^for *p *= 1,6. For the yeast co-expression network, we arrive at the following results. First, *d*^C,[6] ^is highly correlated (> 0.8) with the lower-order GTOM dissimilarities, *d*^T,[0] ^and *d*^T,[1]^. The correlation-based measure *d*^C,[1] ^is highly correlated (0.79) with *d*^T,[2]^. Second, the higher-order GTOM dissimilarities *d*^T,[2] ^and *d*^T,[3] ^show a high correlation of 0.78. Third, two GTOM-based dissimilarities are moderately correlated (< 0.4) if their orders differ by 2 or more. Finally, the frequency distribution of *d*^T,[3] ^is concentrated around 0 while that of the others are concentrated around 1. This illustrates that increasing *m *leads to increased sensitivity but decreased specificity when defining interconnectedness.

#### Hierarchical clustering and GTOM plots

**Figure 4:**
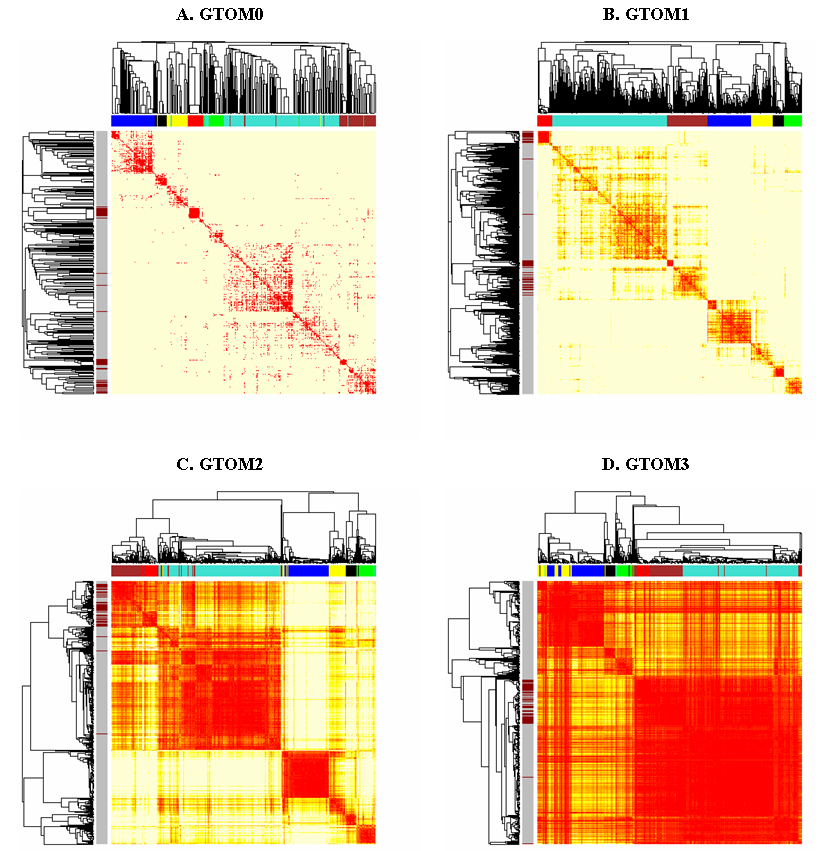
**Topological overlap matrix plots for the yeast gene co-expression network**. **A**. GTOM0 plot. **B**. GTOM1 plot. **C**. GTOM2 plot. **D**. GTOM3 plot. The color bar on the top of each heatmap shows the module assignment obtained from GTOM1. The color bar on the left of each heatmap shows the functional category of the corresponding genes. Dark red indicates the membership to the class 'protein biosynthesis'. Modules are more pronounced in the GTOM2 and GTOM3 plots (larger contrast between the diagonal blocks and off-diagonal blocks). Smaller modules (as diagonal blocks of red) are more visible in GTOM0 and GTOM1 plots whereas larger modules are more respected in GTOM2 and GTOM3 plots. However, GTOM3 leads to excessively large modules and thus the specificity of the modules is compromised. Protein biosynthesis genes are grouped together in the GTOM2 and GTOM3 plots.

In networks involving few nodes, modules can easily be identified by inspecting the network but for large networks involving hundreds of nodes, it is useful to provide a 'reduced' view of the network. For example, one can visualize the topological overlap dissimilarity using classical multi-dimensional scaling plots [27], see the Multi-dimensional Scaling Plots section. Alternatively, it can be useful to visualize the topological overlap dissimilarity matrix [dijT,[m]
 MathType@MTEF@5@5@+=feaafiart1ev1aaatCvAUfKttLearuWrP9MDH5MBPbIqV92AaeXatLxBI9gBaebbnrfifHhDYfgasaacH8akY=wiFfYdH8Gipec8Eeeu0xXdbba9frFj0=OqFfea0dXdd9vqai=hGuQ8kuc9pgc9s8qqaq=dirpe0xb9q8qiLsFr0=vr0=vr0dc8meaabaqaciaacaGaaeqabaqabeGadaaakeaacqWGKbazdaqhaaWcbaGaemyAaKMaemOAaOgabaGaeeivaqLaeiilaWIaei4waSLaemyBa0Maeiyxa0faaaaa@36D4@] directly using a TOM plot. As an example, consider the four GTOM plots corresponding to the zeroth- to third-order GTOM in Figure 4. The dataset used here is the same as the one in Figure 2. Red/yellow indicate low/high values of dijT,[m]
 MathType@MTEF@5@5@+=feaafiart1ev1aaatCvAUfKttLearuWrP9MDH5MBPbIqV92AaeXatLxBI9gBaebbnrfifHhDYfgasaacH8akY=wiFfYdH8Gipec8Eeeu0xXdbba9frFj0=OqFfea0dXdd9vqai=hGuQ8kuc9pgc9s8qqaq=dirpe0xb9q8qiLsFr0=vr0=vr0dc8meaabaqaciaacaGaaeqabaqabeGadaaakeaacqWGKbazdaqhaaWcbaGaemyAaKMaemOAaOgabaGaeeivaqLaeiilaWIaei4waSLaemyBa0Maeiyxa0faaaaa@36D4@. Both rows and columns of dijT,[m]
 MathType@MTEF@5@5@+=feaafiart1ev1aaatCvAUfKttLearuWrP9MDH5MBPbIqV92AaeXatLxBI9gBaebbnrfifHhDYfgasaacH8akY=wiFfYdH8Gipec8Eeeu0xXdbba9frFj0=OqFfea0dXdd9vqai=hGuQ8kuc9pgc9s8qqaq=dirpe0xb9q8qiLsFr0=vr0=vr0dc8meaabaqaciaacaGaaeqabaqabeGadaaakeaacqWGKbazdaqhaaWcbaGaemyAaKMaemOAaOgabaGaeeivaqLaeiilaWIaei4waSLaemyBa0Maeiyxa0faaaaa@36D4@ have been sorted using the hierarchical clustering tree. Since dijT,[m]
 MathType@MTEF@5@5@+=feaafiart1ev1aaatCvAUfKttLearuWrP9MDH5MBPbIqV92AaeXatLxBI9gBaebbnrfifHhDYfgasaacH8akY=wiFfYdH8Gipec8Eeeu0xXdbba9frFj0=OqFfea0dXdd9vqai=hGuQ8kuc9pgc9s8qqaq=dirpe0xb9q8qiLsFr0=vr0=vr0dc8meaabaqaciaacaGaaeqabaqabeGadaaakeaacqWGKbazdaqhaaWcbaGaemyAaKMaemOAaOgabaGaeeivaqLaeiilaWIaei4waSLaemyBa0Maeiyxa0faaaaa@36D4@ is symmetric, the GTOM plot is also symmetric around the diagonal. Since modules are sets of nodes with high (generalized) topological overlap, modules correspond to red squares along the diagonal.

Figure 4 shows that modules are more pronounced and larger with increasing values of *m*. This illustrates that higher values of *m *increase the sensitivity of measuring interconnectedness at the expense of specificity. This is further discussed in the section on the asymptotic behavior of GTOM below. For comparison purposes, a color bar is shown on the top on each GTOM plot. The color bar is ordered by the respective dendrogram and colored by the GTOM1 module assignment (c.f. Figure 2B.) The fact that the module colors stay together for different choices of *m *provides evidence that the module assignment is fairly robust with respect to the dissimilarity measure. One advantage of our proposed general class of dissimilarity measures is that they allow one to verify that module assignment is robust with respect to different network dissimilarities. If there is a strong biological signal, one would hope that the results are robust with respect to different choices of statistical methods.

But a more subtle analysis provides indirect empirical evidence of the usefulness of GTOM2 for module definition. Note that a second color bar is included on the left of the heatmap. Here, dark red indicates the membership to the class 'protein biosynthesis'. Genes that belong to other classes (or are unknown) are depicted by a gray color in the bar. We observe that protein biosynthesis genes are grouped together in the GTOM2 and GTOM3 plots.

#### Multi-dimensional scaling plots

We visualize the dissimilarity measures using classical multi-dimensional scaling (MDS) plots. Classical multidimensional scaling takes as input matrix a dissimilarity matrix (here the GTOM dissimilarity). The result of multi-dimensional scaling are vectors in a low dimensional Euclidean space (here the 2 dimensional Euclidean plane) such that the Euclidean distances between the vectors approximate the dissimilarities. To compute these vectors, an eigenvector problem is solved to find the locations that minimize distortions to the dissimilarity matrix [27].

**Figure 5:**
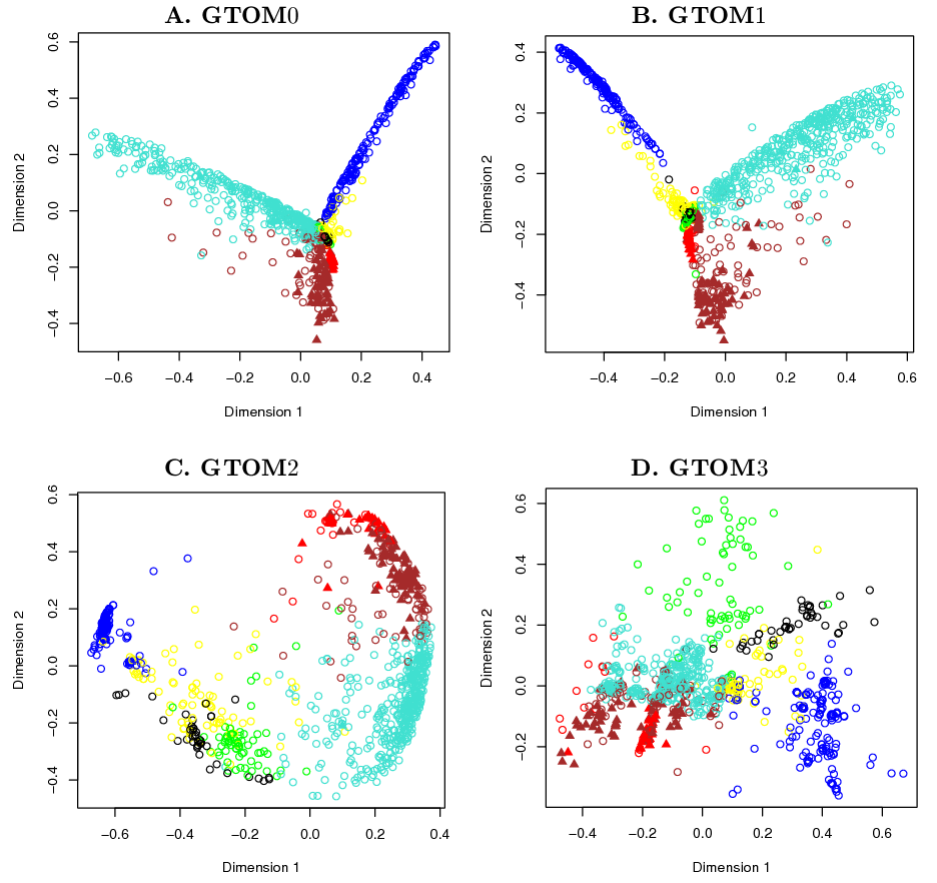
**Multi-dimensional scaling plots of the yeast gene co-expression network**. MDS plots using **A**. GTOM0, **B**. GTOM1, **C**. GTOM2, and **D**. GTOM3. The coloring scheme is used to reflect the 7 modules shown in Figure 2**B **detected by using hierarchical clustering with the GTOM1-based dissimilarity. The symbol '▲' denotes genes that belong to the functional category 'protein biosynthesis'. Genes that belong to other classes are denoted by a '○'. In general, the module assignment is preserved across the different GTOM measures. But the spatial distributions of the points vary to a large extent. Genes in the 'protein biosynthesis' class appear to be closer together.

The MDS plots are shown in Figure 5. All the plots are color-coded according to the modules with respect to GTOM1 depicted in Figure 2. The relative position of the points are well-preserved as we can see that points having the same color are almost always clustered together. Genes that belong to the class 'protein biosynthesis' are depicted by the symbol '▲'. Other genes are denoted by a '○'. Interestingly, almost all 'protein biosynthesis' genes are in the vicinity of each other. The plot using the GTOM1 dissimilarity in Figure 5B shows a more clear separation between the red and brown modules.

We observe from the MDS plots that there is a tendency of consolidation as the order *m *of the GTOM measure increases. This phenomenon can be seen in Figure 5D where GTOM3 is used and a few 'sinks' (points of attraction) have been formed.

### Robustness of the results to network perturbations

To demonstrate that GTOM2 may outperform GTOM1 and GTOM0 in the case of noisy network data, we randomly removed connections in the yeast gene co-expression network. Specifically, we randomly set a proportion *p *= 0, 0.1, 0.25, 0.33, 0.5, 0.67, 0.75, 0.9 of entries in the adjacency matrix to 0. We used the perturbed network to compute the corresponding GTOM*m *measures (for *m *= 0,1,2,3). To quantify the ability of GTOM*m *to separate protein biosynthesis genes (ℬ
 MathType@MTEF@5@5@+=feaafiart1ev1aaatCvAUfKttLearuWrP9MDH5MBPbIqV92AaeXatLxBI9gBamrtHrhAL1wy0L2yHvtyaeHbnfgDOvwBHrxAJfwnaebbnrfifHhDYfgasaacH8akY=wiFfYdH8Gipec8Eeeu0xXdbba9frFj0=OqFfea0dXdd9vqai=hGuQ8kuc9pgc9s8qqaq=dirpe0xb9q8qiLsFr0=vr0=vr0dc8meaabaqaciaacaGaaeqabaWaaeGaeaaakeaaimaacqWFSeIqaaa@377E@) from non-protein biosynthesis genes (Nℬ
 MathType@MTEF@5@5@+=feaafiart1ev1aaatCvAUfKttLearuWrP9MDH5MBPbIqV92AaeXatLxBI9gBamrtHrhAL1wy0L2yHvtyaeHbnfgDOvwBHrxAJfwnaebbnrfifHhDYfgasaacH8akY=wiFfYdH8Gipec8Eeeu0xXdbba9frFj0=OqFfea0dXdd9vqai=hGuQ8kuc9pgc9s8qqaq=dirpe0xb9q8qiLsFr0=vr0=vr0dc8meaabaqaciaacaGaaeqabaWaaeGaeaaakeaaimaacqWFneVtcqWFSeIqaaa@394F@) we defined a measure of separation, which is motivated by the intergroup dissimilarity measures used in average linkage hierarchical clustering. Specifically, we define

GTOMdiff(m)=aℬ−aNℬ     (6)
 MathType@MTEF@5@5@+=feaafiart1ev1aaatCvAUfKttLearuWrP9MDH5MBPbIqV92AaeXatLxBI9gBamrtHrhAL1wy0L2yHvtyaeHbnfgDOvwBHrxAJfwnaebbnrfifHhDYfgasaacH8akY=wiFfYdH8Gipec8Eeeu0xXdbba9frFj0=OqFfea0dXdd9vqai=hGuQ8kuc9pgc9s8qqaq=dirpe0xb9q8qiLsFr0=vr0=vr0dc8meaabaqaciaacaGaaeqabaWaaeGaeaaakeaacqqGhbWrcqqGubavcqqGpbWtcqqGnbqtcqqGKbazcqqGPbqAcqqGMbGzcqqGMbGzcqGGOaakcqWGTbqBcqGGPaqkcqGH9aqpcqWGHbqydaWgaaWcbaacdaGae8hlHieabeaakiabgkHiTiabdggaHnaaBaaaleaacqWFneVtcqWFSeIqaeqaaOGaaCzcaiaaxMaadaqadaqaaiabiAda2aGaayjkaiaawMcaaaaa@500C@

where

aℬ=∑i∈ℬ∑j∈ℬtij[m]|ℬ|2
 MathType@MTEF@5@5@+=feaafiart1ev1aaatCvAUfKttLearuWrP9MDH5MBPbIqV92AaeXatLxBI9gBamrtHrhAL1wy0L2yHvtyaeHbnfgDOvwBHrxAJfwnaebbnrfifHhDYfgasaacH8akY=wiFfYdH8Gipec8Eeeu0xXdbba9frFj0=OqFfea0dXdd9vqai=hGuQ8kuc9pgc9s8qqaq=dirpe0xb9q8qiLsFr0=vr0=vr0dc8meaabaqaciaacaGaaeqabaWaaeGaeaaakeaacqWGHbqydaWgaaWcbaacdaGae8hlHieabeaakiabg2da9maalaaabaWaaabeaeaadaaeqaqaaiabdsha0naaDaaaleaacqWGPbqAcqWGQbGAaeaacqGGBbWwcqWGTbqBcqGGDbqxaaaabaGaemOAaOMaeyicI4Sae8hlHieabeqdcqGHris5aaWcbaGaemyAaKMaeyicI4Sae8hlHieabeqdcqGHris5aaGcbaGaeiiFaWNae8hlHiKaeiiFaW3aaWbaaSqabeaacqaIYaGmaaaaaaaa@532E@

is the average GTOM*m *among protein biosynthesis genes and

**Figure 6:**
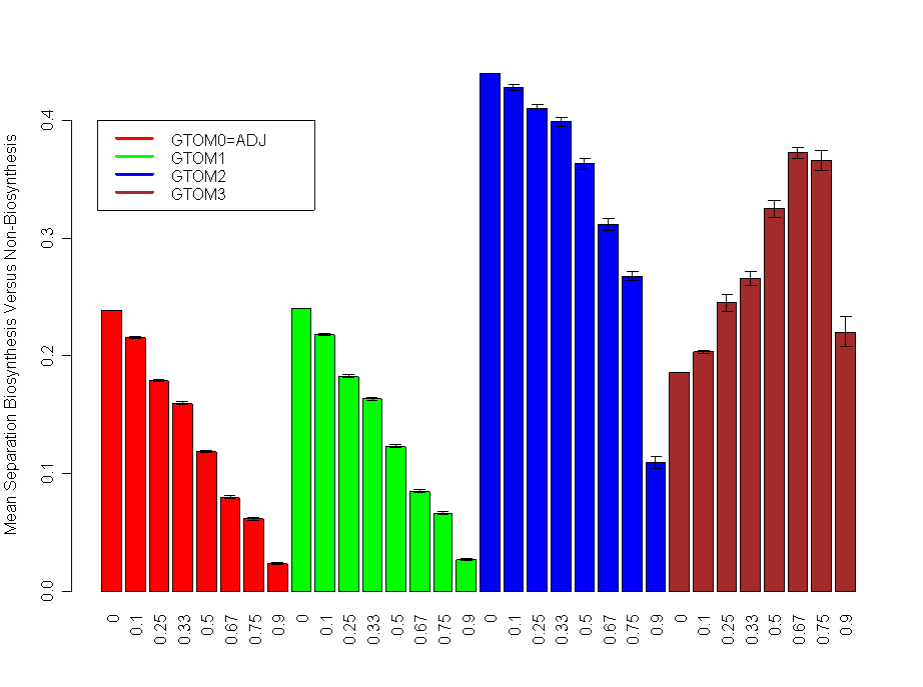
**Separation of protein biosynthesis genes from non-protein biosynthesis genes in perturbed versions of the yeast network**. The average separation (c.f. Eq. 6) is reported for GTOM0 (red), GTOM1 (green), GTOM2 (blue) and GTOM3 (brown). To assess the robustness of the GTOM measures to random deletions, we randomly deleted a proportion *p *of connections (adjacencies) and averaged the results across 20 draws. Note that GTOM2 outperforms the other measures if *p *< 67%. GTOM3 outperforms GTOM2 if more than 67% of adjacencies are deleted. This illustrates that high values of *m *can counter the effect of misspecified (unknown or missing) adjacencies.

aNℬ=∑i∈ℬ∑j∈Nℬtij[m]|ℬ||Nℬ|
 MathType@MTEF@5@5@+=feaafiart1ev1aaatCvAUfKttLearuWrP9MDH5MBPbIqV92AaeXatLxBI9gBamrtHrhAL1wy0L2yHvtyaeHbnfgDOvwBHrxAJfwnaebbnrfifHhDYfgasaacH8akY=wiFfYdH8Gipec8Eeeu0xXdbba9frFj0=OqFfea0dXdd9vqai=hGuQ8kuc9pgc9s8qqaq=dirpe0xb9q8qiLsFr0=vr0=vr0dc8meaabaqaciaacaGaaeqabaWaaeGaeaaakeaacqWGHbqydaWgaaWcbaacdaGae8xdX7Kae8hlHieabeaakiabg2da9maalaaabaWaaabeaeaadaaeqaqaaiabdsha0naaDaaaleaacqWGPbqAcqWGQbGAaeaacqGGBbWwcqWGTbqBcqGGDbqxaaaabaGaemOAaOMaeyicI4Sae8xdX7Kae8hlHieabeqdcqGHris5aaWcbaGaemyAaKMaeyicI4Sae8hlHieabeqdcqGHris5aaGcbaGaeiiFaWNae8hlHiKaeiiFaWNaeiiFaWNae8xdX7Kae8hlHiKaeiiFaWhaaaaa@5B96@

is the average GTOM*m *between protein biosynthesis and non-protein biosynthesis genes. Here, |ℬ
 MathType@MTEF@5@5@+=feaafiart1ev1aaatCvAUfKttLearuWrP9MDH5MBPbIqV92AaeXatLxBI9gBamrtHrhAL1wy0L2yHvtyaeHbnfgDOvwBHrxAJfwnaebbnrfifHhDYfgasaacH8akY=wiFfYdH8Gipec8Eeeu0xXdbba9frFj0=OqFfea0dXdd9vqai=hGuQ8kuc9pgc9s8qqaq=dirpe0xb9q8qiLsFr0=vr0=vr0dc8meaabaqaciaacaGaaeqabaWaaeGaeaaakeaaimaacqWFSeIqaaa@377E@| and |Nℬ
 MathType@MTEF@5@5@+=feaafiart1ev1aaatCvAUfKttLearuWrP9MDH5MBPbIqV92AaeXatLxBI9gBamrtHrhAL1wy0L2yHvtyaeHbnfgDOvwBHrxAJfwnaebbnrfifHhDYfgasaacH8akY=wiFfYdH8Gipec8Eeeu0xXdbba9frFj0=OqFfea0dXdd9vqai=hGuQ8kuc9pgc9s8qqaq=dirpe0xb9q8qiLsFr0=vr0=vr0dc8meaabaqaciaacaGaaeqabaWaaeGaeaaakeaaimaacqWFneVtcqWFSeIqaaa@394F@| denote the total number of protein biosynthesis genes and non-protein biosynthesis genes respectively. The higher the value of GTOMdiff(*m*), the better is the performance of GTOM*m *in this application. For each probability *p*, we averaged the results across 20 perturbed versions of the network. The results in Figure 6 demonstrate that high values of *m *counter the effect of misspecified (missing) adjacencies in this application.

### A simple example

**Figure 7:**
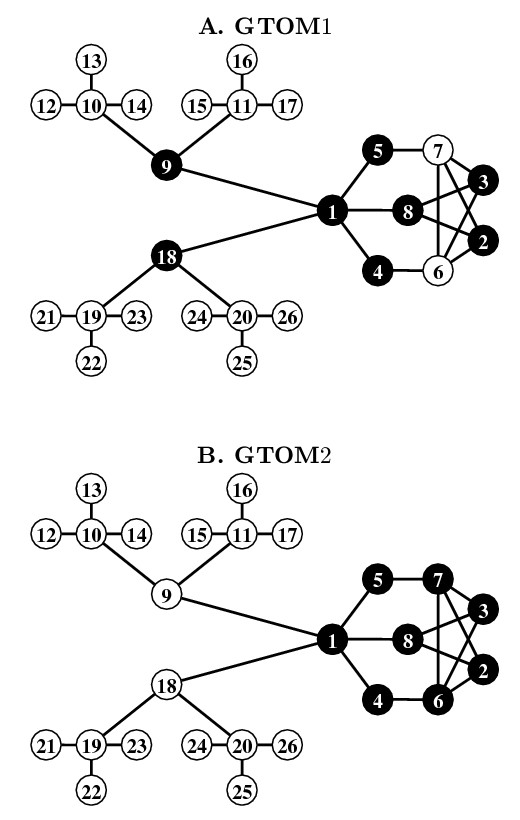
**A simple example where GTOM2 is superior to GTOM1**. GTOM neighborhood of size *S *= 7 around node 1. **A**. GTOM1 neighbors are colored in black. **B**. GTOM2 neighbors are colored in black. Note that GTOM2 detects the 'true' neighborhood (comprised on nodes 1 through 8) while GTOM1 misses nodes 6 and 7.

An example comparing modules detected by the GTOM1 and GTOM2 similarities is given in Figure 7. As a rule of thumb, if many of the nodes in a module are separated by a distance of 1 or 2 from each other, then they form a tight module with respect to the GTOM1 similarity. Likewise, if many of the nodes in a module are separated by a distance of 3 or 4 from each other, then they form a tight module with respect to the GTOM2 similarity.

### The asymptotic behavior of GTOM*m *for large *m*

Here we consider the situation when *m *is larger than or equal to the network diameter, i.e. each pair of nodes can be connected with a path of length ≤ *m*. In this case, |*N*_*m*_(*i*)| = *n *- 1 and |*N*_*m*_(*i*) ∩ *N*_*m*_(*j*)| = *n *- 2 where *n *denotes the network size. Then

tij[m]=n−2+aijn−aij≈1,
 MathType@MTEF@5@5@+=feaafiart1ev1aaatCvAUfKttLearuWrP9MDH5MBPbIqV92AaeXatLxBI9gBaebbnrfifHhDYfgasaacH8akY=wiFfYdH8Gipec8Eeeu0xXdbba9frFj0=OqFfea0dXdd9vqai=hGuQ8kuc9pgc9s8qqaq=dirpe0xb9q8qiLsFr0=vr0=vr0dc8meaabaqaciaacaGaaeqabaqabeGadaaakeaacqWG0baDdaqhaaWcbaGaemyAaKMaemOAaOgabaGaei4waSLaemyBa0Maeiyxa0faaOGaeyypa0ZaaSaaaeaacqWGUbGBcqGHsislcqaIYaGmcqGHRaWkcqWGHbqydaWgaaWcbaGaemyAaKMaemOAaOgabeaaaOqaaiabd6gaUjabgkHiTiabdggaHnaaBaaaleaacqWGPbqAcqWGQbGAaeqaaaaakiabgIKi7kabigdaXiabcYcaSaaa@4870@

when *n *is large. This demonstrates that for sufficiently large values of *m *all pairs of nodes within a connected network component will be highly interconnected. Thus, large and tight modules result when GTOM*m *with large *m *is used as input of a clustering procedure, see Figure 4.

### Choosing the order *m*

How to choose the order *m *is an important question in many applications. While it seems intuitive that the choice of *m *has some relationship to the network diameter, it is unclear to us how to use the network diameter to guide the choice of *m *(other than providing an upper bound).

In general, the optimal choice of *m *will depend on the data quality and the goals of the analysis. Roughly speaking, if the adjacency matrix contains very few errors and if the goal is to determine which nodes are linked to a given node then *m *= 0 is the obvious choice. But if many adjacencies have falsely been set to 0 (since the corresponding connections are unknown) and/or if the goal is to detect possibly longer ranging interactions then relatively large values of *m *may uncover that 2 nodes are interconnected even if the corresponding adjacency is 0.

When an external label is available for at least some of the nodes then one can use it to inform the choice of *m*. For example, when the external node label *y *encodes group membership, then one can choose *m *so that the groups have high within group interconnectedness and low between group interconnectedness. To make this more specific, we assume that there is evidence that the nodes of group 1 are highly interconnected and that they are well separated from nodes of group 0. For example, in our yeast gene co-expression network, we have shown above that the average GTOM*m *measure between protein biosynthesis genes (group 1) is larger than the average GTOM*m *measure between protein biosynthesis genes and non-protein biosynthesis genes (Figure 6).

Analogous to Eq. 6, we define the following measure of mean GTOM difference between the 2 groups

GTOMdiff(m):=∑i≠jtij[m]I(yi=1,yj=1)∑i≠jI(yi=1,yj=1)−∑i≠jtij[m]I(yi=1,yj=0)∑i≠jI(yi=1,yj=0)
 MathType@MTEF@5@5@+=feaafiart1ev1aaatCvAUfKttLearuWrP9MDH5MBPbIqV92AaeXatLxBI9gBaebbnrfifHhDYfgasaacH8akY=wiFfYdH8Gipec8Eeeu0xXdbba9frFj0=OqFfea0dXdd9vqai=hGuQ8kuc9pgc9s8qqaq=dirpe0xb9q8qiLsFr0=vr0=vr0dc8meaabaqaciaacaGaaeqabaqabeGadaaakeaacqqGhbWrcqqGubavcqqGpbWtcqqGnbqtcqqGKbazcqqGPbqAcqqGMbGzcqqGMbGzcqGGOaakcqWGTbqBcqGGPaqkcqGG6aGocqGH9aqpdaWcaaqaamaaqababaGaemiDaq3aa0baaSqaaiabdMgaPjabdQgaQbqaaiabcUfaBjabd2gaTjabc2faDbaaaeaacqWGPbqAcqGHGjsUcqWGQbGAaeqaniabggHiLdGccqWGjbqscqGGOaakcqWG5bqEdaWgaaWcbaGaemyAaKgabeaakiabg2da9iabigdaXiabcYcaSiabdMha5naaBaaaleaacqWGQbGAaeqaaOGaeyypa0JaeGymaeJaeiykaKcabaWaaabeaeaacqWGjbqscqGGOaakcqWG5bqEdaWgaaWcbaGaemyAaKgabeaakiabg2da9iabigdaXiabcYcaSiabdMha5naaBaaaleaacqWGQbGAaeqaaOGaeyypa0JaeGymaeJaeiykaKcaleaacqWGPbqAcqGHGjsUcqWGQbGAaeqaniabggHiLdaaaOGaeyOeI0YaaSaaaeaadaaeqaqaaiabdsha0naaDaaaleaacqWGPbqAcqWGQbGAaeaacqGGBbWwcqWGTbqBcqGGDbqxaaaabaGaemyAaKMaeyiyIKRaemOAaOgabeqdcqGHris5aOGaemysaKKaeiikaGIaemyEaK3aaSbaaSqaaiabdMgaPbqabaGccqGH9aqpcqaIXaqmcqGGSaalcqWG5bqEdaWgaaWcbaGaemOAaOgabeaakiabg2da9iabicdaWiabcMcaPaqaamaaqababaGaemysaKKaeiikaGIaemyEaK3aaSbaaSqaaiabdMgaPbqabaGccqGH9aqpcqaIXaqmcqGGSaalcqWG5bqEdaWgaaWcbaGaemOAaOgabeaakiabg2da9iabicdaWiabcMcaPaWcbaGaemyAaKMaeyiyIKRaemOAaOgabeqdcqGHris5aaaaaaa@9D72@

where the indicator function *I*(·) equals 1 if the condition is satisfied and 0 otherwise. Note that ∑i≠jtij[m]I(yi=1,yj=1)∑i≠jI(yi=1,yj=1)
 MathType@MTEF@5@5@+=feaafiart1ev1aaatCvAUfKttLearuWrP9MDH5MBPbIqV92AaeXatLxBI9gBaebbnrfifHhDYfgasaacH8akY=wiFfYdH8Gipec8Eeeu0xXdbba9frFj0=OqFfea0dXdd9vqai=hGuQ8kuc9pgc9s8qqaq=dirpe0xb9q8qiLsFr0=vr0=vr0dc8meaabaqaciaacaGaaeqabaqabeGadaaakeaadaWcaaqaamaaqababaGaemiDaq3aa0baaSqaaiabdMgaPjabdQgaQbqaaiabcUfaBjabd2gaTjabc2faDbaaaeaacqWGPbqAcqGHGjsUcqWGQbGAaeqaniabggHiLdGccqWGjbqscqGGOaakcqWG5bqEdaWgaaWcbaGaemyAaKgabeaakiabg2da9iabigdaXiabcYcaSiabdMha5naaBaaaleaacqWGQbGAaeqaaOGaeyypa0JaeGymaeJaeiykaKcabaWaaabeaeaacqWGjbqscqGGOaakcqWG5bqEdaWgaaWcbaGaemyAaKgabeaakiabg2da9iabigdaXiabcYcaSiabdMha5naaBaaaleaacqWGQbGAaeqaaOGaeyypa0JaeGymaeJaeiykaKcaleaacqWGPbqAcqGHGjsUcqWGQbGAaeqaniabggHiLdaaaaaa@5D1E@ equals the mean interconnectedness of group 1 nodes and ∑i≠jtij[m]I(yi=1,yj=0)∑i≠jI(yi=1,yj=0)
 MathType@MTEF@5@5@+=feaafiart1ev1aaatCvAUfKttLearuWrP9MDH5MBPbIqV92AaeXatLxBI9gBaebbnrfifHhDYfgasaacH8akY=wiFfYdH8Gipec8Eeeu0xXdbba9frFj0=OqFfea0dXdd9vqai=hGuQ8kuc9pgc9s8qqaq=dirpe0xb9q8qiLsFr0=vr0=vr0dc8meaabaqaciaacaGaaeqabaqabeGadaaakeaadaWcaaqaamaaqababaGaemiDaq3aa0baaSqaaiabdMgaPjabdQgaQbqaaiabcUfaBjabd2gaTjabc2faDbaaaeaacqWGPbqAcqGHGjsUcqWGQbGAaeqaniabggHiLdGccqWGjbqscqGGOaakcqWG5bqEdaWgaaWcbaGaemyAaKgabeaakiabg2da9iabigdaXiabcYcaSiabdMha5naaBaaaleaacqWGQbGAaeqaaOGaeyypa0JaeGimaaJaeiykaKcabaWaaabeaeaacqWGjbqscqGGOaakcqWG5bqEdaWgaaWcbaGaemyAaKgabeaakiabg2da9iabigdaXiabcYcaSiabdMha5naaBaaaleaacqWGQbGAaeqaaOGaeyypa0JaeGimaaJaeiykaKcaleaacqWGPbqAcqGHGjsUcqWGQbGAaeqaniabggHiLdaaaaaa@5D1A@ equals the mean interconnectedness between group 1 and group 0 nodes. Since high values of GTOMdiff(*m*) indicate a good separation between the 2 groups, it is natural to choose *m *as the value that maximizes GTOMdiff(*m*). Obviously, this criterion for choosing *m *only works if prior data allow one to define the external label *y*.

## Discussion

Several measures that keep track of shared 1 step neighbors have been proposed in the literature, e.g. [28]. Here, we propose a natural generalization of the widely used topological overlap matrix. This class of new measures is constructed by keeping track of the number of *m*-step neighbors that a pair of nodes share. The GTOM similarity measure is normalized to take on values in the unit interval. A corresponding dissimilarity measure can be defined by subtracting the GTOM similarity from 1.

While we find it a worthwhile goal for future research to develop statistical or heuristic criteria for choosing *m*, we find that a main advantage of GTOM*m *is that it allows one to assess the robustness of network analysis results. In many applications (e.g. module definition, neighborhood analysis), it will be worthwhile to show that the results are relatively robust with respect to *m *since this indicates that the biological signal is strong. While we present applications where non-standard choices of *m *lead to superior results, we have found in several other (unreported) applications that the results are robust with respect to *m *= 0,1, 2. By randomly deleting network adjacencies in the yeast gene co-expression network application, we have shown that large values of *m *can counter the effect of misspecified (missing) adjacencies. GTOM*m *becomes uninformative if *m *is larger than the network diameter. Thus, GTOM*m *will be useful in networks with moderate or large 'degree of separation' (average path length between any pair of nodes). Since biological networks tend to have low diameters [29], we expect that low values of *m *will be preferable in most applications. But we have provided two real data applications where *m *= 2 is preferable over *m *= 1. In general, the GTOM measures with lower orders *m *will be useful for discovering small modules while those with higher orders favor the discovery of larger modules.

A limitation of our approach is that it is only defined for unweighted networks, i.e. the entries of the adjacency matrix should be 0 or 1. Another limitation is that we only consider the topological overlap between 2 nodes. A multi-node extension of the GTOM1 measure is presented in [30].

## Conclusion

The generalized topological overlap measure can serve as a filter for countering the effect of spurious or missing connections. The order *m *of the topological overlap measure can serve as a tuning parameter for interconnectedness that trades off sensitivity versus specificity. Since different orders of *m *probe different neighborhoods, adjusting *m *allows the user to consider network modules at different 'zoom' levels. We provide additional Materials and Methods as well as the statistical software code, a tutorial along with customized R functions, and the accompanying data files at the web page [31]. Thus, the reader should be able to reproduce all of our findings.

## Methods

### Topological overlap matrix

The topological overlap of two nodes reflects their similarity in terms of the commonality of the nodes they connect to. Note that in an unweighted network, the number of shared neighbors of nodes *i *and *j *is given by ∑_*u*≠*i,j *_*a*_*iu *_*a*_*uj*_. Ravasz *et al*. [1] define the topological overlap measure *t*_*ij *_as follows

tij={lij+aijmin⁡{ki,kj}+1−aijif i≠j1if i=j     (7)
 MathType@MTEF@5@5@+=feaafiart1ev1aaatCvAUfKttLearuWrP9MDH5MBPbIqV92AaeXatLxBI9gBaebbnrfifHhDYfgasaacH8akY=wiFfYdH8Gipec8Eeeu0xXdbba9frFj0=OqFfea0dXdd9vqai=hGuQ8kuc9pgc9s8qqaq=dirpe0xb9q8qiLsFr0=vr0=vr0dc8meaabaqaciaacaGaaeqabaqabeGadaaakeaacqWG0baDdaWgaaWcbaGaemyAaKMaemOAaOgabeaakiabg2da9maaceqabaqbaeqabiGaaaqaamaalaaabaGaemiBaW2aaSbaaSqaaiabdMgaPjabdQgaQbqabaGccqGHRaWkcqWGHbqydaWgaaWcbaGaemyAaKMaemOAaOgabeaaaOqaaiGbc2gaTjabcMgaPjabc6gaUjabcUha7jabdUgaRnaaBaaaleaacqWGPbqAaeqaaOGaeiilaWIaem4AaS2aaSbaaSqaaiabdQgaQbqabaGccqGG9bqFcqGHRaWkcqaIXaqmcqGHsislcqWGHbqydaWgaaWcbaGaemyAaKMaemOAaOgabeaaaaaakeaacqqGPbqAcqqGMbGzcqqGGaaicqWGPbqAcqGHGjsUcqWGQbGAaeaacqaIXaqmaeaacqqGPbqAcqqGMbGzcqqGGaaicqWGPbqAcqGH9aqpcqWGQbGAaaaacaGL7baacaWLjaGaaCzcamaabmaabaGaeG4naCdacaGLOaGaayzkaaaaaa@656E@

where *l*_*ij *_= ∑_*u*≠*i,j *_*a*_*iu *_*a*_*uj*_, *k*_*i *_= ∑_*u*≠*i *_*a*_*iu*_. An advantage of the quantity 1 - *a*_*ij *_in the denominator is that it prevents the denominator from becoming 0 when the connectivities (degrees) *k*_*i *_and *k*_*j *_are 0. Since *a*_*ij *_≤ 1, one can easily show that *l*_*ij *_= ∑_*u*≠*i,j *_*a*_*iu *_*a*_*uj *_≤ ∑_*u*≠*i *_*a*_*iu *_- *a*_*ij *_= *k*_*i *_- *a*_*ij*_. It follows that *l*_*ij *_≤ min(*k*_*i *_- *a*_*ij*_, *k*_*j *_- *a*_*ij*_) and that the numerator of *t*_*ij *_is smaller than the denominator, i.e. 0 ≤ *t*_*ij *_≤ 1.

We remark that the definition of TOM given in [1] is slightly different from Eq. (7):

(*l*_*ij *_+ *a*_*ij*_)/min{*k*_*i*_, *k*_*j*_}. In a personal communication with E. Ravasz, the definition in Eq. (7) is preferred, which is also given in the online supporting material of [1]. The inclusion of the term *a*_*ij *_in the numerator makes *t*_*ij *_explicitly depend on the existence of a direct link between the two nodes in question.

### An algorithm for computing GTOM

In this subsection, we present computational formulas for *T*^[*m*]^. In this subsection, we assume that the diagonal of *A *has been set to 0. Then the *ij*-th entry of the matrix power *A*^*m *^counts the number of paths of length *m *connecting nodes *i *and *j *[32]. But the paths are not necessarily geodesic and may contain cycles. Then the matrix *S*^[*m*] ^≡ [sij[m]
 MathType@MTEF@5@5@+=feaafiart1ev1aaatCvAUfKttLearuWrP9MDH5MBPbIqV92AaeXatLxBI9gBaebbnrfifHhDYfgasaacH8akY=wiFfYdH8Gipec8Eeeu0xXdbba9frFj0=OqFfea0dXdd9vqai=hGuQ8kuc9pgc9s8qqaq=dirpe0xb9q8qiLsFr0=vr0=vr0dc8meaabaqaciaacaGaaeqabaqabeGadaaakeaacqWGZbWCdaqhaaWcbaGaemyAaKMaemOAaOgabaGaei4waSLaemyBa0Maeiyxa0faaaaa@34E3@]:= *A *+ *A*^2 ^+ ... + *A*^*m *^counts how many distinct paths of length smaller than or equal to *m *connect each pair of nodes. Thus, we have *N*_*m*_(*i*) = {*j *≠ *i *|sij[m]
 MathType@MTEF@5@5@+=feaafiart1ev1aaatCvAUfKttLearuWrP9MDH5MBPbIqV92AaeXatLxBI9gBaebbnrfifHhDYfgasaacH8akY=wiFfYdH8Gipec8Eeeu0xXdbba9frFj0=OqFfea0dXdd9vqai=hGuQ8kuc9pgc9s8qqaq=dirpe0xb9q8qiLsFr0=vr0=vr0dc8meaabaqaciaacaGaaeqabaqabeGadaaakeaacqWGZbWCdaqhaaWcbaGaemyAaKMaemOAaOgabaGaei4waSLaemyBa0Maeiyxa0faaaaa@34E3@ > 0}. If we define a binary matrix *B*^[*m*] ^to be

bij[m]={1if sij[m]>0 and i≠j,0otherwise,
 MathType@MTEF@5@5@+=feaafiart1ev1aaatCvAUfKttLearuWrP9MDH5MBPbIqV92AaeXatLxBI9gBaebbnrfifHhDYfgasaacH8akY=wiFfYdH8Gipec8Eeeu0xXdbba9frFj0=OqFfea0dXdd9vqai=hGuQ8kuc9pgc9s8qqaq=dirpe0xb9q8qiLsFr0=vr0=vr0dc8meaabaqaciaacaGaaeqabaqabeGadaaakeaacqWGIbGydaqhaaWcbaGaemyAaKMaemOAaOgabaGaei4waSLaemyBa0Maeiyxa0faaOGaeyypa0ZaaiqabeaafaqaaeGacaaabaGaeGymaedabaGaeeyAaKMaeeOzayMaeeiiaaIaem4Cam3aa0baaSqaaiabdMgaPjabdQgaQbqaaiabcUfaBjabd2gaTjabc2faDbaakiabg6da+iabicdaWiabbccaGiabbggaHjabb6gaUjabbsgaKjabbccaGiabdMgaPjabgcMi5kabdQgaQjabcYcaSaqaaiabicdaWaqaaiabb+gaVjabbsha0jabbIgaOjabbwgaLjabbkhaYjabbEha3jabbMgaPjabbohaZjabbwgaLjabcYcaSaaaaiaawUhaaaaa@5EBE@

then *N*_*m*_(*i*) ≡ {*j *≠*i*|bij[m]
 MathType@MTEF@5@5@+=feaafiart1ev1aaatCvAUfKttLearuWrP9MDH5MBPbIqV92AaeXatLxBI9gBaebbnrfifHhDYfgasaacH8akY=wiFfYdH8Gipec8Eeeu0xXdbba9frFj0=OqFfea0dXdd9vqai=hGuQ8kuc9pgc9s8qqaq=dirpe0xb9q8qiLsFr0=vr0=vr0dc8meaabaqaciaacaGaaeqabaqabeGadaaakeaacqWGIbGydaqhaaWcbaGaemyAaKMaemOAaOgabaGaei4waSLaemyBa0Maeiyxa0faaaaa@34C1@ = 1}. To obtain the number of shared *m*-step neighbors, |*N*_*m*_(*i*) ∩ *N*_*m*_(*j*)|, we simply take the inner product of the *i*-th and the *j*-th columns of *B*^[*m*] ^which can be obtained from the matrix (*B*^[*m*]^)^2 ^= [|*N*_*m*_(*i*) ∩ *N*_*m*_(*j*)|] because of the symmetry of *B*^[*m*]^. In particular, |*N*_*m*_(*i*)| is given by the *i*-th diagonal entry of (*B*^[*m*]^)^2^. These values can then be used to compute *T*^[*m*] ^using formula (3). It is worth repeating that the formulas in this subsection assume that the diagonal of the adjacency matrix is 0. Since matrix multiplication is computationally expensive, the computation of *S*^[*m*] ^may be sped up using the formula *A*(*S*^[*m*-1]^+ *I*).

### Using hierarchical clustering for module detection

By using the topological overlap measure as an input of the average linkage hierarchical clustering procedure, we define modules as discrete branches of the clustering tree (e.g. Figure 2). As in all hierarchical clustering analysis, it is a judgement call where to cut the tree branches. When detecting modules using hierarchical clustering, we use GTOM plots to aid the choice of the dendrogram's height cutoff (see the Results section). Thus the modules are found by inspection: a height cutoff value is chosen in the dendrogram such that some of the resulting branches correspond to the discrete diagonal blocks (modules) in the GTOM plot. The robustness of the module definition with respect to the height cut-off can be explored using our online R software tutorial.

### Yeast gene co-expression network construction

Two genes in our co-expression network are linked if they are highly correlated across the samples. To construct the gene co-expression networks from the microarray data [22], we first select the 4000 yeast genes having the highest variance across the microarray samples. Then we calculated all possible pairwise Pearson correlations for the 4000 genes across the microarrays. Because microarray data can be noisy and the number of samples is often small, absolute values of the correlations were thresholded using a relatively large hard threshold of *τ *= 0.7. This threshold corresponds to a significance level of *p *= 8.7 × 10^-8 ^(Fisher's correlation test) and leads to an *approximate *scale-free topology as described in [33]. Such a topology implies the existence of 'hub genes' [34] and the robustness to random perturbations [35] which are biologically desirable properties. The topology of the yeast network data is further discussed in [3].

## Authors' contributions

Both authors jointly developed the methods, implemented them, and wrote the article.
